# Difficulties in Establishing “Truth” Conditions in the Assessment of Addictive Smartphone Use in Young Adults

**DOI:** 10.3390/ijerph19010358

**Published:** 2021-12-30

**Authors:** Javier García-Manglano, Claudia López-Madrigal, Charo Sádaba-Chalezquer, Cecilia Serrano, Olatz Lopez-Fernandez

**Affiliations:** 1 Institute of Culture and Society, University of Navarra, 31008 Pamplona, Spain; 2 School of Education and Psychology, University of Navarra, 31008 Pamplona, Spain; clopez.30@unav.es; 3 School of Communication, University of Navarra, 31008 Pamplona, Spain; csadaba@unav.es; 4 Department of Sociology, Catholic University of the Sacred Heart, 20123 Milan, Italy; anacecilia.serranonunez@unicatt.it; 5 Health Research Institute, Hospital Universitario Fundación Jiménez Díaz, 28040 Madrid, Spain

**Keywords:** problematic smartphone use, smartphone addiction, cutoff points, smartphone addiction scale, Spanish SAS-SV, psychological distress, depression, anxiety, stress, social media use

## Abstract

The smartphone revolution has placed powerful, multipurpose devices in the hands of youth across the globe, prompting worries about the potential negative consequences of these technologies on mental health. Many assessment tools have been created, seeking to classify individuals into problematic and non-problematic smartphone users. These are identified using a cutoff value: a threshold, within the scale range, at which higher scores are expected to be associated with negative outcomes. Lacking a clinical assessment of individuals, the establishment of this threshold is challenging. We illustrate this difficulty by calculating cutoff values for the Short Version of the Smartphone Addiction Scale (SAS-SV) in 13 Spanish-speaking samples in 11 countries, using common procedures (i.e., reliability, validity, ROC methodology). After showing that results can be very heterogeneous (i.e., they lead to diverse cutoff points and rates of addiction) depending on the decisions made by the researchers, we call for caution in the use of these classifications, particularly when researchers lack a clinical definition of true addiction—as is the case with most available scales in the field of behavioral addictions—which can cause an unnecessary public health alert.

## 1. Introduction

Over the past decade, as handheld digital devices with constant internet access became ubiquitous, worries about the consequences of problematic smartphone use on youth development have multiplied. Even though touchscreen handheld phones were introduced in the early 2000s, it was the release of the iPhone and the Android OS in 2007 that initiated the smartphone revolution; the number of smartphones in active use in the United States doubled from 50 to 100 million between 2009 and 2011, and doubled again from 100 to 200 million between 2011 and 2014. These numbers were estimated to be near 300 million in 2019 [1] in a population of around 330 million. Globally, the number of active smartphones (mobile subscriptions) grew from 724 million in 2011 to 6401 million in 2021 [2].

The smartphone changed communications in important ways. By providing full portability, it opened the possibility of constant connectivity, anytime and anywhere. By creating an ecosystem for mobile applications it expanded functionality from voice calls to all sorts of activities—from calendar to videogames, from entertainment to note-taking. With time, dropping smartphone prices broke income barriers, bringing constant connectivity to places where desktops and laptops had not yet arrived, e.g., low-income areas and groups. All of this made it increasingly common to see powerful devices in the hand of adolescents and teens. By 2018, 95% of US teens had a smartphone, with little class variability, and the percentage was 93% among teens in households with an income lower than $30 K, and 97% among teens in households with an income higher than $75 K [3].

It soon became apparent that mobile phones could have implications for well-being and mental health, and the 2010s witnessed the development of a plethora of instruments (inventories, scales) aimed at classifying individuals by their problematic mobile phone use, in different languages and cultures [4,5]. 

These classifying tools are varied in their perspective and goals; some take a more behavioral approach, seeking to assess ‘problematic’ use, while others take a more clinical approach and claim to be able to identify signs of ‘addictive’ use. In this paper, we do not aim to determine whether the behavioral or the clinical approach is more convenient. Instead, we explore, using one of the most popular assessment tools available to the scientific community, the complexities of establishing clear classifications of individuals in this area of research, sounding a call for caution in the use of these assessment tools. 

### 1.1. One Construct, Many Tools

One of the first literature reviews on mobile phone abuse or addiction was undertaken by Pedrero and colleagues [6]. This study identified some important scientific problems, including the conceptual vagueness of the concepts used to refer to the excessive use of mobile phones, the disparity in the adoption of diagnostic criteria, and the numerous psychometric instruments for the assessment of these concepts. They recorded estimated prevalence ranges between 0 and 38%, depending on the scale used (out of the 15 scales tested between 2005 and 2011, almost all without robust psychometric tests and replication) and the characteristics of the population (usually adolescents and young adults from Asia).

In a recent systematic review of available problematic phone use scales, Harris et al. [7] analyzed the properties of 78 such instruments—all of them created between 2004 and 2019. These scales were of three types, most of them attempting to measure problematic smartphone use (70 scales), some exploring smartphone use motivations and attitudes (six scales), and a few assessing smartphone use frequency (three scales, one of which was also included in the previous group). The authors identified a series of problems in the development of smartphone use scales, such as the exclusive use of self-reported data (even when seeking to assess criterion-related validity), or the frequent absence of test-retest reliability coefficients (available in only 10 out of the 78 scales) to assess the temporal stability of the instruments. Thus, the study of problematic mobile phone use is still rife with potential measurement problems. 

### 1.2. The Smartphone Addiction Scale

The Smartphone Addiction Scale (SAS) is an instrument created with a clinical perspective, which is gaining higher recognition over the MPPUS, possibly because it is more adapted to the current smartphones rather than mobile phones. Both have shown similar psychometric properties, but the SAS is the most representative scale to measure present problematic smartphone use for two reasons according to a recent review [8]: (i) it is highly cited across numerous research fields showing it remains influential; (ii) among the ten scales used in the empirical study, this was the one which captured the largest variance from the construct shared across all measures. Our choice of instrument, however, does not imply that we endorse clinical approaches in general, or this scale in particular—instead, we use the SAS because of its popularity and evidence-based support: it has been cited over a thousand times in less than 10 years, and it has been translated into multiple languages covering the immense majority of the world population, from the most populous languages, like Chinese, Spanish, English, Arabic, French, German, Italian or Portuguese, to lesser used languages, like Malay or Persian. See Table 1.

The SAS was originally developed in 2013 by Min Kwon and colleagues at the Catholic University of Korea, in Seoul. The development of the scale began with a 48-item questionnaire assessing seven dimensions of addiction (i.e., daily-life disturbance, disturbance of reality, positive anticipation, withdrawal, cyberspace-oriented relationship, overuse, and tolerance), and a 7-item visual analogue scale in which respondents subjectively rated their own addiction to smartphones [17]. The same authors developed a short version of the SAS (SAS-SV) with 10 items in the Korean language, which was particularly suited for adolescents [9]. 

Kwon and colleagues went on to calculate a cutoff point dividing adolescents between those exhibiting symptoms of ‘addiction’ in the use of their smartphones and those making a moderate use. Notably, this process included the clinical assessment of 150 adolescents (90 boys, 60 girls) by professional psychologists; in this assessment, 16.6% of the boys and 26.6% of the girls exhibited symptoms of addiction, tolerance, and withdrawal. These clinical assessments were used as true conditions for a ROC analysis [18], determining cutoff points of 31 for men and 33 for women [9]. We used the SAS-SV as the basis for our analysis, in its Spanish adaptation [10], which in turn suggested a unique cutoff value of 32 for both boys and girls. 

### 1.3. The SAS-SV and Concurrent Outcomes

Over the years, researchers have explored the association between problematic smartphone use and a variety of variables, from socioeconomic characteristics to mental health issues. We review some of those associations, with particular attention to studies using the SAS-SV and the four outcomes we will use in our analysis—see below.

#### 1.3.1. Psychological Distress: Anxiety, Depression, Stress

Due to their prevalence and interference in daily activities, anxiety, depression, and stress have become the gold standard in the assessment of psychological distress. Multiple studies have found significant associations between addictive or problematic smartphone use as measured by the SAS-SV, and anxiety [19,20,21,22,23,24,25], depression [21,25,26,27,28,29], and stress [24,30,31,32]. 

Similar results were found in studies that assessed problematic smartphone use with different scales, such as the Bergen Social Networking Addiction Scale, BSNAS [28,33], the Problematic Mobile Phone Use Scale, PMPUS [34,35], the Technology Use Questionnaire [36], the Problematic Use of Mobile Phone, PUMP [37], and the Smartphone Addiction Inventory, SPAI [38].

A systematic review of 23 peer-reviewed papers [39] explains that people with mental health issues try to compensate for their emotional difficulties with an overuse of the smartphone to alleviate their negative emotions; a technique which turns out to be ineffective. Because psychopathological symptoms are related to difficulties in regulating emotions and in enacting adaptive strategies, problematic use of technology can relate to the presence of psychopathology [27]. However, more longitudinal studies are needed to establish directionality between depression, anxiety, stress, and smartphone use. 

#### 1.3.2. Screen Time and Social Media

Studies have also established a link between higher scores of problematic smartphone use and time spent on screens and social media [20,25,27,40,41,42,43,44,45]. According to displacement theory [46], time spent on social media can displace important activities, such as face-to-face interactions or sleep [47]. Similar research shows that time spent on screens might be detrimental to one’s own well-being by altering or displacing positive lifestyle habits, such as sports and hobbies [29,48,49,50,51].

However, small or non-significant relations have also been reported between smartphone use and both the SAS-SV scores [52] and other problematic smartphone use scales [36,37,53,54,55]. These studies contend that time spent on social media is not necessarily a predictor of poor mental health when we account for other processes, such as use of motivations and socio-contextual variables that impact both mental health and time spent on devices [56]. Additionally, youth use multiple devices for multiple purposes (e.g., gaming, texting, entertainment, work, study), some of which are positive, so the amount of screen time spent alone might be a poor proxy for problematic smartphone use.

In sum, the aim of this paper is to warn about a common practice in the field of smartphone addiction research: the proposal and use of cutoff points to classify users as addicted or non-addicted, while lacking a clinical assessment of addiction to use as ‘true’ condition against which to compare the statistical instrument (e.g., scale, assessment tool). We sound our warning by replicating common practice, using one of the most widely used smartphone addiction scales (SAS-SV) across 13 Spanish-speaking samples. Even though we calculate cutoff points, our goal is not to select the best one among them, but to show that picking one among many possible cutoff points is a difficult task that should be carried out in a more careful, transparent way than what we find in most studies to date. 

## 2. Materials and Methods

### 2.1. Participants and Procedure

Data for this study were collected mostly between March and September of 2020, except for one sample which was collected in March of 2021. The data collection effort began in the context of a project devised in 2019. Ethical approval was obtained from the Ethics Committee of the University of Navarra (2020.087) in early 2020. Researchers went into the field in late February 2020, before the global COVID-19 pandemic. Data collection was interrupted by the health crisis in March 2020, when 624 telephone surveys had been completed. In this extraordinary situation, the team of researchers decided that the original data collection effort in Spain would be interrupted until the lockdown was lifted. Starting in May 2020, as limitations waned, researchers went back into the field to collect the remaining 576 telephone surveys (completing the sample Spain 1 on 18 June 2020). This Spanish sample of 1200 youth aged 18 to 22 (Spain 1) was recontacted a year later (March 2021) for a follow-up telephone interview (Spain 2—retention rate was 87% of the original sample, and the remaining 13% was replaced with youth with similar sociodemographic characteristics). These two telephone interviews are the only nationally representative samples in our study. Even though these are panel data, for the purposes of this study we use the two samples separately.

In March 2020, when the pandemic started, the team decided to expand the project internationally, inviting academic peers in 10 Spanish-speaking countries to contribute with online surveys. This resulted in the collection of 11 cross-sectional convenience samples (using the same questionnaire) in Argentina, Chile, Colombia, Ecuador, El Salvador, Guatemala, México, Perú, Spain (Spain 3), Uruguay, and Venezuela. 

Table 2 provides sample sizes and some demographic data on each sample. To increase comparability across samples, we selected sub-samples of individuals ages 18 to 29 years old in each country. The sample sizes vary between 212 in Argentina and 1200 in Spain. Online surveys produced convenience samples, which resulted in an over-representation of women which was particularly acute in Colombia (80.1% women) and Argentia (77.8% women). 

Average ages ranged from 20 years old in Spain 1 to almost 24 years old in Ecuador. Respondents with higher levels of education were over-represented in all non-nationally representative samples, particularly in Argentina and Chile, where about half of the sample had reached higher-education levels. 

### 2.2. Instruments

The online survey used for the present study comprised three sections: (a) sociodemographics, (b) the Spanish SAS-SV version previously validated by Lopez-Fernandez (2017), and (c) scales with related constructs. The socio-demographic variables included were gender, age, and country. 

The SAS-SV included 10 items rated on from 1 (‘strongly disagree’) to 6 (‘strongly agree’), giving a score between 10 and 60 in which the highest score shows the maximum presence of ‘smartphone addiction’. It has originally shown content and concurrent validity and internal consistency (Cronbach’s Alpha: 0.91). Items include situations, such as ‘missing planned work due to smartphone use’, ‘being told by people around me that I use my smartphone too much’ or ‘feeling impatient and fretful when not holding my smartphone’ (for a complete list of the items, in English and Spanish, see Appendix A). The scales of related constructs were the following:

- Psychological distress. Anxiety, depression, and stress through the DASS-21 scale [57]; following the authors’ manual [58]. It is a self-reporting scale with 21 items, seven per category: depression (e.g., loss of self-esteem and low mood), anxiety (e.g., fear anticipation of negative outcomes), and stress (e.g., state of hyper-arousal and low-frustration tolerance). Each item is measured with a 4-point severity/frequency rating scale from 0 (‘did not apply to me at all’) to 3 (‘applied to me very much, or most of the time’), with higher scores representing more severe levels of psychological distress in the last week. The original Cronbach Alphas are 0.91, 0.84 and 0.90 for the subscales of depression, anxiety, and stress, respectively. We flagged respondents with severe or extremely severe scores in each subscale: anxiety (a value of 8 or more), depression (11 or more), and stress (13 or more).
- Time in social media. Following common practice [16,59,60], we flagged individuals with more than 4 h of self-reported daily use of social media (net of the time spent in direct communication, which was collected as a separate question).

### 2.3. Analysis Plan

Adapting and validating scales involves a series of steps, each of which includes, in turn, several small but potentially relevant decisions. The goal of this analysis is to unpack those decisions and to illustrate their relevance, particularly regarding the classification of participants into categories below and above some theoretically relevant cutoff point. We focus on decisions concerning the estimation of a threshold to classify participants into ‘addicted’ (note that when we classify individuals as ‘addicted’ we are not making a clinical assessment, but simply following the SAS-SV (Smartphone Addiction Scale) rationale. We are of the belief that the addiction framework is not appropriate for this sort of scale, and will refer to ‘problematic use’ in all cases, except when we explicitly refer to the output of the SAS-SV. In any case, we will always use quotation marks to indicate that ‘addicted’ is a scale-derived construct, not necessarily a diagnosis or an accurate description of reality) or ‘non-addicted’ individuals. We use all 13 samples to illustrate the difficulties inherent in this process; the Spanish version of the SAS-SV was applied in all samples. We used Stata 16 for our analyses.

Following the original SAS-SV paper [17], we took three evaluating steps (omitting those directly related to item selection):

1. Reliability: evaluates internal consistency, i.e., the degree to which the items that make up a scale behave consistently with respect to the construct being evaluated. This is evaluated using the overall Cronbach Alpha of SAS-SV in each sample.
2. Concurrent validity (criterion-related): refers to the extent to which a scale correlates with indicators (criteria) that are theoretically expected to be associated with it. We tested Pearson’s correlations between the SAS-SV and four external criteria: anxiety, depression, stress, and excessive social media.
3. Cutoff point calculation: a process that involves the following sub-steps:
  - Determine a ‘true condition’, a criterion capable of identifying ‘addicted’ smartphone users. The gold standard would be to carry out a clinical evaluation of each individual. However, this is expensive and seldom done (in the case of the SAS-SV and the nine language versions presented in Table 1, only the original scale by Kwon and colleagues used clinical evaluations to determine true smartphone ‘addiction’). Lacking a clinical evaluation, researchers use external criteria which are expected to be associated with the scale, to distinguish between truly ‘addicted’ individuals (what we will call ‘problematic profiles’ below). These external criteria include, for example, addiction symptoms from the DSM-IV [61,62], lack of self-control [63], subjective feeling of smartphone addiction [32] symptoms of depression and anxiety [21,64], or correlation with other internet addiction scales [65].
  - Compare SAS-SV scores with the true condition above, calculating true positives (TP: ‘addicted’ individuals who were correctly classified as such), false positives (FP: people classified ‘addicted’ by the SAS-SV who are not truly ‘addicts’), true negatives (TN: subjects who are not ‘addicted’ and were correctly classified as such), and false negatives (FN: ‘addicts’ who are classified as not having any problem with their smartphone use). From these values, one calculates the sensitivity [TP/(TP + FN)] and the specificity [TN/(TN + FP)] of the SAS-SV scale.
  - Calculate a cutoff point: using receiver operator characteristics (ROC) curve methodology, look for the cutoff value that maximizes the area under this (ROC) curve (AUC). This is given by the highest Youden Index among all possible combinations of true conditions and cutoff points.

This is a complex process that involves a series of decisions that might sway the results one way or another. Particularly problematic is, we argue, the definition of true conditions when researchers lack expert clinical evaluations of the outcome being assessed (in our case, smartphone ‘addiction’). In the remainder of this paper, we use our 13 Spanish-speaking samples to show that, depending on the decisions taken along the way, researchers can obtain very heterogeneous results. Here we present only results from the general sample of individuals under the age of 30.

## 3. Results

### 3.1. Reliability and Concurrent Validity

The SAS-SV exhibits a strong degree of reliability across samples, with Cronbach Alphas above 0.8, with Argentina being (α = 0.81) the lowest and Ecuador (α = 0.87) the highest. See Table 3.

With respect to concurrent validity (also called criterion-related validity), we observe significant correlations between SAS-SV scores and four of the criteria that the literature generally associates with problematic smartphone use: anxiety, depression, stress, and daily time in social media. Correlations are as high as 0.51 between SAS-SV scores and severe stress in Venezuela; the lowest scores (still statistically significant) are with social media use, ranging from 0.15 in Colombia to 0.34 in the Spain 1 sample. 

Generally, this implies that the SAS-SV is a reliable estimate, one that covaries significantly with measures of psychological distress and with social media screen time. Most adaptations of the scale (and most uses and adaptations of similar tools designed to measure problematic smartphone use) stop here, assuming that this is enough to guarantee a proper use of the scale. Below, we argue that here is where things might get messier; a series of decisions, and differences across samples, can still lead to very highly heterogeneous results. 

### 3.2. Difficulties in Establishing a Cutoff Point Points for Each Sample

After assessing reliability and concurrent validity, we build 15 potential problematic profiles, by combining (individually, in pairs, and in groups of three and four) four variables commonly associated with problematic smartphone use: anxiety, depression, stress, and time in social media. We flag individuals with severe symptoms in the problematic smartphone use related constructs, such as psychological distress and time on social media.

Table 4 presents five of the fifteen potential problematic profiles. It shows the proportion of individuals in our samples who exhibit severe rates of anxiety, depression, or stress, as well as those using social media for more than 4 h every day. We note that the prevalence of these conditions, taken one by one, among youth aged 18 to 29, is relatively high; most proportions are between 20% and 40%. 

The last column in Table 4 shows the percentage of youth scoring high in all four of the criteria; a very strict condition, met by less than 10% of youth in all countries. To make our case as conservative as possible, we will use this most stringent criterion to determine whether a participant is truly problematic in his use of the smartphone (‘addicted’, in SAS-SV terminology). We considered adding other criteria, such as subjective sense of control in the use of the smartphone, or youth’s attachment styles; however, these were discarded from our final models due to weak correlation with other indicators and with SAS-SV (results available upon request).

The next step, after determining the true condition of the individuals in our sample (i.e., participants who show problematic symptomatology potentially associated with detrimental smartphone use), was to calculated *sensitivity* (the ability to correctly identify a person with smartphone addiction, i.e., to minimize false negatives) and *specificity* (the ability to correctly identify a person with a healthy level of smartphone use, i.e., to minimize false positives). These calculations were repeated for each sample, under each of the 15 potential problematic profiles described above, providing 195 cutoff points for the determination of ‘smartphone addiction’ using the SAS-SV (data available upon request).

These cutoff points are extremely diverse, ranging from a low of 21 to a high of 45; it is important to notice that these cutoff points would all be statistically possible; theoretically, they would only differ in the way they define the true condition, lacking a clinical assessment of individuals. To illustrate this disparity, the low and high cutoff points correspond to the following scenarios:

- In Ecuador, if we determined the true condition with ‘social media’ as the only criterion (individuals using it for longer than 4 h per day), the statistically ideal cutoff point would be 21 (out of 60). This would lead to the conclusion that 69% of Ecuadorians are ‘addicted’ to their smartphone.
- In Venezuela, if we determined the true condition as those with severe symptoms of anxiety and depression, who also use social media more than 4 h a day, the statistically ideal cutoff point would be 45. We would conclude that only 7.8% of Venezuelans are ‘addicted’ to the smartphone.

In between these two extremes, we find all possible combinations of criteria. Even though, looking at these results, one would be tempted to disregard the criteria used to define a true condition as theoretically inappropriate, these are not uncommon criteria; they all have been used in previous research, sometimes with the SAS-SV scale, sometimes with another one of the other 78 scales assessing problematic smartphone use reviewed by Harris et al. [7].

### 3.3. Two Possible Rules to Choose Cutoff Points

Given that our 13 datasets and 15 potential definitions of problematic smartphone use profiles produced up to 195 potential cutoff points, we need criteria to discriminate between such a diverse set of scenarios. 

The usual rule to pick one cutoff point among many, within the same sample, is to maximize the area under the ROC curve, which is the same as picking the cutoff point with the highest Youden Index [9]. The main advantage of this rule is that it provides the cutoff point with the best balance between sensitivity and specificity, i.e., the cutoff point that minimizes misclassification of individuals; the allocation of ‘addicted’ participants in the ‘non-addicted’ category, and vice-versa. Its main problem is that it might lead to a definition of different true conditions across samples. One scale, like the SAS-SV, would classify individuals in the ‘addicted’ and ‘non-addicted’ categories based on diverse criteria; for example, in one sample the true condition would be defined by having severe anxiety and excessive use of social media (i.e., Chile), while in another sample the true condition would be defined by having severe anxiety, depression, and stress, and using social media extensively (i.e., Spain 2, Colombia, Ecuador, El Salvador, México). 

Table 5 further illustrates this point; it represents the criterion that maximizes the Youden Index, together with the recommended cutoff value, and the proportion of the general population that would be defined as ‘addicted’, in each of our 13 samples. We find up to five possible criteria: (1) SM: severe stress, excessive use of social media; (2) AM: severe anxiety, excessive social media use; (3) DSM: severe depression, severe stress, excessive social media use; (4) ASM: severe anxiety, severe stress, excessive social media use; and (5) ADSM: severe anxiety, severe depression, severe stress, and excessive social media use (this was the most common solution, found in five of our thirteen samples). 

Following this rule, our resulting cutoff points would vary between 26 in Guatemala and 38 in Argentina; the percentage of ‘addicted’ individuals would vary in the opposite direction (as expected, since higher cutoff values mean more stringent definitions of ‘addiction’), with 52.7% of ‘addicted’ youth in Guatemala, and only 16.5% of ‘addicted’ youth in Argentina. 

A second possible rule, beyond picking the true condition with the highest Youden Index in each sample, is to pick a theoretically relevant definition of the true condition and apply it to all samples, regardless of whether it leads you to pick the option with the highest Youden Index. Even if this rule would lead to instances in which the classification error is higher than it might otherwise be, it has the advantage of unifying the criteria for the definition of a true condition, making it possible to compare results sensibly across samples. 

Table 6 illustrates this rule, by showing the solutions provided by the most stringent definition of the true problematic condition: a profile in which the individual presents symptoms of severe anxiety, severe depression, severe stress, as well as excessive use of social media (over 4 h per day). If we were to follow this second rule (a common definition of the true condition), cutoff values would range from 26 in Guatemala to 40 in Argentina, leading us to determine that 52.7% of Guatemalan youth are ‘addicted’ to their smartphone, compared to only 14.2% of Argentinians. 

One final option to be considered would be the creation of a common cutoff point across all Spanish-speaking countries, by combining all samples into one. This would perhaps facilitate comparability across samples, but it might also mask important cultural differences. We explored this option. Using the total sample of 6937 youth aged 18 to 39, the results provided an overall SAS-SV cutoff score of 33, regardless of the rule employed (highest Youden Index, or using ADSM as the true condition). This is not far from the original Korean study which established a cutoff value of 31 for men and 33 for women [9] and the Spanish adaptation which established a common cutoff point of 32 for both men and women [10]. This common cross-sample cutoff point would result in an overall rate of 23% ‘addicted’ youth, but the percentages would vary by sample as follows: 23% in Spain 1, 20% in Spain 2, 18% in Spain 3, 20% in Argentina, 28% in Chile, 26% in Colombia, 22% in Ecuador, 33% in El Salvador, 22% in Guatemala, 21% in Mexico, 27% in Perú, 23% in Uruguay, and 27% in Venezuela. 

## 4. Discussion

The smartphone revolution has put powerful, multipurpose devices in the hands of youth across the globe, leading many to wonder about the potential negative consequences of these technologies on mental health. As a result, the research community has provided many competing assessment tools, many of which attempt to identify individuals with a higher risk of developing unhealthy habits in the use of their smartphones. These individuals, it is argued, can be identified because they cross some theoretically meaningful quantifiable threshold; a diagnostic cutoff score within the range of possible values given by a scale.

The present study aimed to illustrate the difficulty in establishing ‘true’ conditions and objective cutoff points, by applying the Spanish SAS-SV to 13 different Spanish-speaking samples. We explored the psychometric properties of this scale, reliability (Cronbach’s Alpha) and concurrent validity (i.e., based on the variety of criteria), and the resulting cutoff points aimed at estimating users with problematic use of their smartphone within Hispanophone countries.

Regarding the main psychometric properties, the Spanish SAS-SV obtained similar internal consistencies, such as the original one ([9]), i.e., 0.81 to 0.87 (vs. 0.91 of the original SAS-SV and the 0.88 Spanish SAS-SV [10]). This therefore shows the stability of this measure alongside time and space, even during the pandemic.

Concerning criterion-related validity, the SAS-SV shows significant correlations with anxiety, depression, and stress, across all thirteen samples (most of them in the 0.3 to 0.5 range). Regarding social media time, the scale also presents significant associations in all samples. Thus, the SAS-SV proves a reliable estimate which varies along with measures of psychological distress and is associated with social media time. Similar findings have been observed regarding depression and anxiety as predictors of smartphone addiction [64], although other mental health constructs also seem to be related, such as insomnia, social phobia, or loneliness [66]. Problematic smartphone use has been associated with a variety of outcomes, aside from those explored here. Those include personality traits, such as impulsiveness and the ability to self-control [64,67,68]. An individual’s prevalent attachment style can also be related to her use of the smartphone; secure attachment prevents excessive use [69,70], whereas avoidant and insecure attachment increases excessive use [71,72,73], in what Billieux and colleagues [74] described as the reassurance-seeking pathway to problematic smartphone use. 

However, the most original contribution of the present study is to compute all possible cutoff scores resulting from the 15 smartphone problematic profiles derived from all possible combinations between the four related constructs (i.e., depressive, anxious, stressed, and spending excessive time on social media), in all 13 samples. To the best of the authors’ knowledge, the field of technological behavioral addiction lacks an evidence-based, consistent strategy to compute cutoff values of a cross-cultural validated scale, such as the SAS-SV. This study is a first attempt to explore the complexity of this problem. 

We obtained numbers that vary enormously across Spanish countries. The cutoff points range from 26 to 40, resulting in proportions of ‘addicted’ individuals that vary between 14% and 58%. Even when we try to impose the same decisions across all samples, we get proportions of ‘addicted’ youth that can be as low as 18% and as high as 33%.This might correspond to reality; there is no reason to believe that all countries must have similar proportions of youth engaging in problematic smartphone use. Nonetheless, it proves our point: the decisions and rules deployed by researchers might be highly consequential for the results obtained. 

These findings provide a warning and a silver lining. First, the warning is that, given that most researchers do not have (as we do) alternative samples to assess the robustness of their results, they should be very clear about their decisions, making sure that their choices are not only methodologically correct, but also theoretically grounded—as recent literature reviews have stated repeatedly [5,6,7]. We also find, in our data, a silver lining. When we compare the final results obtained by applying the two decision rules (highest Youden; common true condition) sample by sample, we obtain the same cutoff points and percentages of ‘addicted’ youth in 11 out of our 13 samples; different cutoff values are found only in Argentina (38 vs. 40, respectively) and Peru (36 vs. 33). This means that, while one could not guarantee similar results when applying different criteria and rules, there is the possibility that, all in all, they tend to produce converging results. In this case, it would be up to the researchers to justify their decision only in the few cases in which there are dissimilar results for the same sample or country. 

This research is not without limitations. First, only two of our samples are nationally representative; all other samples are online convenience samples, with some of them having been taken in countries where internet connectivity is limited in regard to large segments of the population. Thus, most of our samples over-represent individuals with higher education and social class, with a prevalence of women over men. Second, we did not carry out objective clinical assessments, which is the ideal tool to determine the true ‘addicted’ condition of individuals; this limitation is present in the study design, given that most scale adaptations and validations do not use this gold standard either. Our goal was, precisely, to warn about the heterogeneity of results that one might obtain when building that ‘true’ condition lacking a clinical assessment. Absent of this gold standard, researchers could try different specifications and explore the robustness of their results. In the end, decisions can be both clinically and methodologically sound, empirically robust, and theoretically meaningful.

Establishing cutoff points, therefore, needs to have a gold standard, which should emerge from clinical assessment with a methodological basis. As stated in the title, this clinical control is difficult because researchers have no gold standard to compare with the result of SAS-SV. Each participant’s clinical assessment is necessary in future studies to reach the cutoff point for smartphone addiction subsequently. 

Finally, there is no reason to expect that cutoff points will remain stable over time. When it comes to the behavior of youth and rapidly evolving technologies, one should expect changes in the types and motivations of use of smartphones. Thus, cutoff points might prove poor classification tools after only a few years, as smartphones become better tools to carry out more and more useful daily tasks. For this reason, cutoff points should be reassessed and updated with some frequency. 

## 5. Conclusions

In this paper, we use the SAS-SV. Available in at least 10 languages covering most of the world population, the SAS-SV is one of the most widely used assessment tools in this area of research. We deployed a similar questionnaire, containing the SAS-SV and other relevant outcomes, in 13 samples in 11 Spanish-speaking countries, including Spain and 10 Latin-American nations. With these data, we illustrated the difficulties in establishing a true condition to evaluate and estimate a cutoff point for scales seeking to classify individuals, by their smartphone use, as ‘addicted’ or ‘not-addicted’ to their devices. 

We were able to show how and why the decisions made by the researcher matter in the conclusions one draws about the prevalence of problematic smartphone uses across samples and countries. In consequence, we recommend transparency when reporting the decisions taken in the process of classifying individuals into categories. Hence our warning: researchers seeking to validate or adapt a scale or seeking to establish cutoff points for existing scales that measure clinical conditions, should either find a way to assess individuals with clinical assessment tools, or be very careful about the decisions they make to compensate for the lack of that gold standard—a clinical assessment. It seems to us that many studies have perhaps been unaware of these difficulties, applying tools across countries, languages, and samples without a careful consideration of the consequences of their decisions, not to mention other cultural and socioeconomic issues which must also be addressed, but fall outside the scope of this paper. 

It is our hope that this study will help assess the adaptation and validation of other scales beyond the SAS-SV, and revisit some of the evidence drawn from studies that resorted uncritically to cutoff points to classify individuals in strict categories, leading perhaps to the misclassifications of people as ‘addicted’ or ‘problematic’.

## Figures and Tables

**Table 1:** Characteristics of the SAS-SV in nine different language adaptations.

Language	Reference	*N*	% Female	Mean Age	Cutoff Value (Gender)	Percentage “Addicted” (Gender)	Clinical Assessment
Korean	Kwon et al., 2013 [9]	540	36.5	14.5	31 (men) 33 (women)	20.6% 16.6% (men) 26.6% (women)	Yes
Spanish (Spain)	López-Fernández, 2017 [10]	281	80.1	25.61	32 (unique) ^a^	12.8% 15.2% (men) 10.2% (women)	Symptoms ^b^
French (Belgium)	López-Fernández, 2017 [10]	144	68.8	29.11	32 (unique) ^a^	21.5% 20% (men) 22% (women)	Symptoms ^b^
Portuguese (Brazil)	Andrade et al., 2020 [11]	718 ^c^	63.5	22.1 35.2	31 (men) 33 (women)	39.4% 37.7% (men) 40.4% (women)	Symptoms ^b^
Moroccan	Sfendla et al., 2018 [12]	310	50.6	23.1	31 (men) 33 (women)	55.8% (sex differences not specified)	No
German (Switzerland)	Haug et al., 2015 [13]	1519	51.8	17	31 (men) 33 (women)	16.9% 14.1% (men) 19.4% (women)	No
Italian	De Pasquale et al., 2017 [14]	633	55.4	18	31 (men) 33 (women)	NA	No
Chinese	Tsun Luk et al., 2018 [15]	3211	54.7	43.3	31 (men) 33 (women)	37.7% 35.3% (men) 40.3% (women)	No
Serbian	Nikolik et al., 2021 [16]	323	69.0	21	31 (men) 33 (women)	19.5% 14% (men) 22% (women)	No

^a^ Calculated new cutoff points; all other adaptations used the same cutoff points given by Kwon et al. (2013). ^b^ The symptoms assessed were loss of control, disruption, disregard, withdrawal, preoccupation, and tolerance. Each had a score of 1 to 6, and the average scores had to be equal to or greater than 4 points to be considered as present. ^c^ The sample was part university students (*N* = 387, M age = 22.1) part adults (*N* = 331, M age = 35.2).

**Table 2:** Samples, collection information, demographic characteristics, and SAS-SV scores.

Sample	*N* Total Sample	*N* Sample Age < 30	Fieldwork (Start–End) [dd/mm/yy]	% Female	Average Age (sd)	% with Higher Education	SAS-SV Scores ^2^ Mean (sd)
Spain 1 ^1^	1200	1200	27/02/20–18/06/20	50.0	20 (1.5)	13.8	27 (9.8)
Spain 2 ^1^	1200	1200	1/03/21–16/04/21	50.0	21 (1.5)	13.2	25.2 (9.9)
Spain 3	986	393	30/03/20–29/07/20	60.8	22.6 (3.3)	39.7	26.3 (9.6)
Argentina	547	212	31/03/20–20/06/20	77.8	23.2 (2.9)	50.9	29.7 (9.4)
Chile	504	180	1/04/20–15/09/20	65.6	22.5 (4.2)	41.1	31.16 (10.6)
Colombia	770	276	31/03/20–3/09/20	80.1	22.4 (3.8)	39.1	30.6 (10.3)
Ecuador	766	433	1/04/20–30/07/20	67.2	23.8 (3.2)	48.0	28.5 (10.2)
El Salvador	641	408	1/04/20–3/06/20	58.8	21.8 (3.2)	27.5	32.2 (10.5)
Guatemala	1619	792	2/04/20–29/08/20	54.2	22.5 (3.5)	42.7	28.7 (9.4)
México	1084	475	30/03/20–3/08/20	69.9	22.7 (3.3)	43.6	29.3 (10.2)
Perú	1083	720	2/02/20–24/09/20	65.3	22 (3.5)	37.2	30.1 (9.9)
Uruguay	876	405	1/04/20–29/09/20	70.4	22.5 (3.6)	36.8	29.3 (10.1)
Venezuela	624	243	8/04/20–23/06/20	71.6	22.2 (3.4)	28.0	30.4 (10.6)

^1^ Nationally representative samples administered via phone interview, with quota sampling of the Spanish territory and a margin of error of ±2.5% for a sample size of 1200; the rest are online convenience samples; we set a minimum sample size of 500 per country, for the general population, to guarantee some statistical power with margins of error circa ±5%. ^2^ SAS-SV scores range from 6 to 60.

**Table 3:** Reliability and concurrent validity of SAS-SV.

Sample	Reliability (Cronbach α)	Pearson’s Correlation with			
		Anxiety	Depression	Stress	Social Media Use
Spain 1	0.83	0.42 ***	0.36 ***	0.42 ***	0.34 ***
Spain 2	0.84	0.36 ***	0.32 ***	0.40 ***	0.31 ***
Spain 3	0.85	0.35 ***	0.36 ***	0.39 ***	0.31 ***
Argentina	0.81	0.23 ***	0.24 ***	0.32 ***	0.30 ***
Chile	0.86	0.39 ***	0.47 ***	0.47 ***	0.31 ***
Colombia	0.84	0.30 ***	0.40 ***	0.38 ***	0.15 *
Ecuador	0.87	0.41 ***	0.37 ***	0.41 ***	0.24 ***
El Salvador	0.86	0.28 ***	0.37 ***	0.39 ***	0.21 ***
Guatemala	0.83	0.27 ***	0.30 ***	0.33 ***	0.20 ***
México	0.85	0.36 ***	0.39 ***	0.40 ***	0.19 ***
Perú	0.86	0.41 ***	0.42 ***	0.45 ***	0.23 ***
Uruguay	0.84	0.37 ***	0.40 ***	0.44 ***	0.32 ***
Venezuela	0.85	0.45 ***	0.40 ***	0.51 ***	0.23 ***

* *p* < 0.05; *** *p* < 0.001.

**Table 4:** Five profiles of problematic youth: potential criteria for the definition of a true condition for ‘addictive’ smartphone use.

	Percentage of Young People with Symptoms of Each Construct				
Sample	Severe Anxiety	Severe Depression	Severe Stress	Using Social Media 4 + Hours	Problematic in All Four Criteria
Spain 1	28.5	17.2	18.8	22.2	4.0
Spain 2	24.1	15.6	19.8	18.5	2.5
Spain 3	22.4	22.9	22.1	14.8	4.6
Argentina	19.3	18.4	23.1	21.7	1.9
Chile	38.3	33.3	38.3	32.8	7.2
Colombia	37.3	27.9	34.4	33	9.4
Ecuador	39.5	25.2	28.2	37.4	8.3
El Salvador	37.5	33.3	33.3	31.4	7.1
Guatemala	29.8	24.0	26.3	26.4	5.3
Mexico	30.3	23.4	29.3	30.5	5.5
Peru	41.5	33.9	35.8	32.5	9.0
Uruguay	20.5	22.5	24.0	23.0	4.0
Venezuela	37.9	26.3	32.1	38.7	9.1

**Table 5:** True conditions, cutoff values, and percentage of ‘addicted’ youth when highest Youden Index is used as the decision rule.

Sample	Youden Index	True Condition ^1^	Cutoff Value	% Addicted
Spain 1	0.462	DSM	30	36.0
Spain 2	0.485	ADSM	31	23.2
Spain 3	0.546	ASM	31	27.2
Argentina	0.659	SM	38	16.5
Chile	0.469	AM	35	37.8
Colombia	0.418	ADSM	28	58.3
Ecuador	0.432	ADSM	35	24.3
El Salvador	0.445	ADSM	36	34.6
Guatemala	0.318	SM	26	52.7
México	0.566	ADSM	32	35.0
Perú	0.393	DSM	36	28.8
Uruguay	0.656	DSM	34	32.6
Venezuela	0.478	SM	33	36.2

^1^ True conditions are combinations of: A: severe anxiety; D: severe depression; S: severe stress; M: more than 4 h per day on social media.

**Table 6:** Youden Index, cutoff values, and percentage of ‘addicted’ youth, when the same definition is given for the true problematic condition across samples.

Sample	Youden Index	True Condition ^1^	Cutoff Value	% Addicted
Spain 1	0.458	ADSM	30	36.0
Spain 2	0.485	ADSM	31	23.2
Spain 3	0.508	ADSM	31	27.2
Argentina	0.601	ADSM	40	14.2
Chile	0.398	ADSM	35	37.8
Colombia	0.418	ADSM	28	58.3
Ecuador	0.432	ADSM	35	24.3
El Salvador	0.445	ADSM	36	34.6
Guatemala	0.297	ADSM	26	52.7
México	0.566	ADSM	32	35.0
Perú	0.378	ADSM	33	34.2
Uruguay	0.637	ADSM	34	32.6
Venezuela	0.320	ADSM	33	36.2

^1^ True conditions are combinations of: A: severe anxiety; D: severe depression; S: severe stress; M: more than 4 h per day on social media.

## Data Availability

Data is not publicly available due to ongoing analyses. However, the data presented in this study can be available upon request to the corresponding author.

## Appendix A

**Table A1:** Complete list of the items in English and Spanish of the Smartphone Addiction Scale Short Version (SAS-SV).

	English	Spanish
1	Missing planned work due to smartphone use	Debido al uso del smartphone he perdido tareas/actividades/trabajos/etc. previamente planificados
2	Having a hard time concentrating in class, while doing assignments, or while working due to smartphone use	Debido al uso del smartphone he tenido problemas de concentración (en clase, en el trabajo, etc.), mientras hacía mis tareas (deberes, etc.) o mientras trabajaba
3	Feeling pain in the wrists or at the back of the neck while using a smartphone	Debido al uso del smartphone he sentido dolor en alguna de mis muñecas o detrás del cuello (por ejemplo, en la nuca), etc.
4	Will not be able to stand not having a smartphone	No puedo estar sin mi smartphone
5	Feeling impatient and fretful when I am not holding my smartphone	Me siento impaciente e inquieto cuando no tengo mi smartphone
6	Having my smartphone in my mind even when I am not using it	Tengo mi smartphone en mente incluso cuando no lo uso
7	I will never give up using my smartphone even when my daily life is already greatly affected by it	No dejaré de usar mi smartphone incluso si mi vida cotidiana está realmente afectada por éste
8	Constantly checking my smartphone so as not to miss conversations between other people on Twitter or Facebook	Estoy comprobando constantemente mi smartphone para no perderme conversaciones con otras personas en las redes sociales (como Twitter, Facebook, etc.)
9	Using my smartphone longer than I had intended	Uso mi smartphone más de lo que había previsto inicialmente
10	The people around me tell me that I use my smartphone too much	La gente de mi alrededor me dice que uso demasiado mi smartphone
